# The characteristics and activities of child and adolescent mental health services in Italy: a regional survey

**DOI:** 10.1186/1471-244X-12-7

**Published:** 2012-01-30

**Authors:** Laura Pedrini, Giovanni Colasurdo, Stefano Costa, Michela Fabiani, Linda Ferraresi, Emilio Franzoni, Francesca Masina, Renato Moschen, Vittoria Neviani, Stefano Palazzi, Roberto Parisi, Antonia Parmeggiani, Antonio Preti, Cosimo Ricciutello, Marco BL Rocchi, Davide Sisti, Antonio Squarcia, Stefano Trebbi, Donatella Turchetti, Paola Visconti, Andrea Tullini, Giovanni de Girolamo

**Affiliations:** 1 IRCCS Centro San Giovanni di Dio-Fatebenefratelli, Brescia-Italy; 2 AUSL di Forlì, UONPIA, Forlimpopoli-Italy; 3 UOS Psichiatria e Psicoterapia età evolutiva, Ospedale Maggiore, Bologna-Italy; 4 AUSL di Reggio Emilia, UONPIA, Reggio Emilia-Italy; 5 AUSL di Modena, UONPIA, Sassuolo (MO)-Italy; 6 A.O. Universitaria Orsola-Malpighi, Bologna-Italy; 7 AUSl di Cesena, UONPIA, Cesena-Italy; 8 U.O. NPI Ospedaliera-Psicopatologia dell'Adolescenza e U.O. NPI Ospedaliera-Neurologia RIMINI-Italy; 9 Il Nespolo, Ospedale Privato Villa Igea, Modena-Italy; 10 AUSl di Ferrara, UONPIA, Ferrara-Italy; 11 AUSL di Piacenza, UONPIA, Piacenza-Italy; 12 IRCCS Institute of Neurological Sciences and Department of Neurological Sciences, University of Bologna; 13 Department of Psychology, University of Cagliari-Italy; 14 AUSL of Imola, UONPIA, Imola (BO)-Italy; 15 Institute of Biomathematics, University of Urbino-Italy; 16 AUSL di Parma, UONPIA, Parma-Italy; 17 AUSL di Bologna, UONPIA, Monzuno (BO)-Italy; 18 AUSL di Ravenna, UONPIA, Ravenna-Italy; 19 AUSL di Bologna, Ospedale Maggiore, Bologna-Italy; 20 AUSL di Rimini, UONPIA, Rimini-Italy

**Keywords:** Child and Adolescent Mental Health Services (CAMHS), Process of care, Adolescence, Child Psychiatry

## Abstract

**Background:**

To date, no studies have assessed in detail the characteristics, organisation, and functioning of Child and Adolescent Mental Health Services (CAMHS). This information gap represents a major limitation for researchers and clinicians because most mental disorders have their onset in childhood or adolescence, and effective interventions can therefore represent a major factor in avoiding chronicity. Interventions and mental health care are delivered by and through services, and not by individual, private clinicians, and drawbacks or limitations of services generally translate in inappropriateness and ineffectiveness of treatments and interventions: therefore information about services is essential to improve the quality of care and ultimately the course and outcome of mental disorders in childhood and adolescence.

The present paper reports the results of the first study aimed at providing detailed, updated and comprehensive data on CAMHS of a densely populated Italian region (over 4 million inhabitants) with a target population of 633,725 subjects aged 0-17 years.

**Methods:**

Unit Chiefs of all the CAMHS filled in a structured 'Facility Form', with activity data referring to 2008 (data for inpatient facilities referred to 2009), which were then analysed in detail.

**Results:**

Eleven CAMHS were operative, including 110 outpatient units, with a ratio of approximately 20 child psychiatrists and 23 psychologists per 100,000 inhabitants aged 0-17 years. All outpatient units were well equipped and organized and all granted free service access. In 2008, approximately 6% of the target population was in contact with outpatient CAMHS, showing substantial homogeneity across the eleven areas thereby. Most patients in contact in 2008 received a language disorder- or learning disability diagnosis (41%). First-ever contacts accounted for 30% of annual visits across all units. Hospital bed availability was 5 per 100,000 inhabitants aged 0-17 years.

**Conclusion:**

The percentage of young people in contact with CAMHS for mental disorders is in line with those observed in previous epidemiological studies. The overall number of child psychiatrists per 100,000 inhabitants is one of the highest in Europe and it is comparable with the most well equipped areas in the US. This comparison should be interpreted with caution, however, because in Italy, child psychiatrists also treat neurological disorders. Critical areas requiring improvement are: the uneven utilisation of standardised assessment procedures and the limited availability of dedicated emergency services during non-office hours (e.g., nights and holidays).

## Background

Over the last 20 years in Italy, several studies have surveyed the characteristics and functioning of adult mental health services, which underwent a radical reform in 1978 (Law no. 180) [1]; on the contrary, no studies to date have examined the characteristics, organisation, and functioning of Child and Adolescent Mental Health Services (CAMHS). Surprisingly, even in Europe and internationally there is a total lack of health services studies providing detailed information about the characteristics, functioning and activity data of CAMHS. This information gap represents a major limitation for researchers and clinicians because: (i) most mental disorders have their onset in childhood or adolescence [2], effective interventions can therefore represent a major factor in avoiding chronicity; (ii) childhood and adolescence are especially vulnerable phases of individual development, and services aimed at preventing and managing age-specific risk factors and related distress are thus of great public health relevance; (iii) registry and epidemiological data in Italy show that up to 8% of the child and adolescent population may meet criteria for mental disorders [3]; and lastly, (iv) interventions and mental health care are delivered by and through services, and not by individual, private clinicians, and drawbacks or limitations of services generally translate in inappropriateness and ineffectiveness of treatments and interventions. Therefore, information about services is essential to improve the quality of care, and ultimately the course and outcome of mental disorders in childhood and adolescence.

In Italy CAMHS are operative throughout the country within 146 Italian Health Districts, and each CAMHS is responsible for all child and adolescent mental health care within defined catchment area. In most regions (including Emilia-Romagna, where this survey took place) CAMHS are a component of Departments of Mental Health, which provide child, adult, and geriatric mental health care as well addiction treatment.

A specific characteristic of Italian CAMHS is that they are responsible for the prevention, assessment, and treatment of both psychiatric and neurological conditions; this is a model framework that may be of interest to other nations struggling with similar problems. Yet, the survey described in this paper specifically focused on mental disorders and their treatment. More detailed information on neurodevelopmental disorders was not within the scope of this study.

This paper reports the results of the first study carried out in an European country, in a large area with more than 4 millions inhabitants, aimed at assessing in detail physical characteristics, staffing arrangements, activities, and care patterns of inpatients and outpatients CAMHS.

## Methods

The study was conducted in Emilia-Romagna, a densely populated region in Northern Italy, with approximately 4 million inhabitants and 633,725 children and adolescents aged 0-17 years. The area's population size is similar to that of some European countries (e.g., Denmark, Slovakia, Finland), and overall the region has a larger population than 8 of Europe's 27 countries.

All 11 regional CAMHS agreed to participate in the study. Each CAMHS appointed a study coordinator, who organised and supervised data collection throughout the project's various phases.

### Data collection

Two specific forms were developed for the CAMHS survey: the "Outpatient Facility form (OFF)" and the "Inpatient Facility Form (IFF)". Both were based on the 'Facility Form', which had been previously used in a national survey of acute psychiatric facilities in Italy [1]; these draft forms were revised several times and then pilot-tested in 2 units.

The final version of the OFF has 156 items divided into the following 7 sections: general information, environmental characteristics, technical equipment and service organisation, available treatments, collaboration with other health services and with schools, staff, procedures and activity data refering to 2008.

On the other hand, the final version of the IFF has 62 items divided into the following six sections: general information, environmental characteristics, staff, procedures, collaboration with child and adolescent outpatient units, and activity data refering to 2009.

The OFF was filled in by the Chief Physician of each CAMHS unit. A specific query was then run in the service's computer-based registry in order to complete the "activity data refering to 2008" section.

After data collection, a thorough quality control check was conducted, first locally throughout the region, and then centrally. Monthly meetings were organised among all the study coordinators to verify data quality (missing data and misprints). This revision process lasted approximately 6 months and ensured a rather low percentage of missing data (< 5%).

### 2.1. Statistical analysis

Descriptive statistics were used to summarise facility characteristics and activity data. Means, standard deviations, and data ranges were calculated for quantitative variables; bivariate statistics were performed for categorical variables using two-way tables with Cramer's V association coefficient, whereas for quantitative variables scatterplots with linear regression were performed. All analyses were conducted using SPSS, version 13.0 for Windows.

## Results

### Outpatient CAMHS: Environmental characteristics and technical equipment

Overall, the region's 11 CAMHS comprised 110 outpatient Units, 43 of which were larger Units ("*CNPIAs*"-*Centri di Neuropsichiatria dell'Infanzia e dell'Adolescenza*, i.e. Child and Adolescence Neuropsychiatry Centres) and 67 of which were simple Outpatients Unit; all but one were public facilities.

All 110 participating units had a specific catchment area: most (N = 72; 65%) had a catchment area of up to 50,000 inhabitants, and 22 (20%) units were linked to a catchment area of more than 100,000 inhabitants.

The target population of 0-17 years was less than 10,000 in 80 units (72.7%), 43 of which (39.1% of the total amount) had fewer than 3,000 inhabitants in the age range examined. The remaining 30 (27%) units had a catchment area of more than 10,000 resident children and adolescents.

Approximately half of the units (for which facility history information was available) (N = 99; 90%) had been built over the last four decades (N = 41; 41.4%); 29 units (29.3%) had been built before 1950 (some had been fully refurbished). In most cases (N = 87, 79.1%), the units were hosted in a building together with other public health services. Twenty-five units (22.7%) had a separate dedicated access, and a dedicated reception area was present in 39 units (35.5%).

Forty units (36.4%) had a dedicated meeting room not used for clinical activities. Soundproofing, an important privacy feature in outpatient settings, was ensured in 74 units (67.3%).

All but 17 units had at least one dedicated room for neuropsychiatric exams; most units (101; 91.8%) had at least one room for other clinical activities (most frequently, for clinical psychologists).

In general, the participating units were equipped with games and other materials to entertain children (N = 106; 96.4%), and all had most of the technical instruments required for conducting medical exams (e.g. diaphanoscope, ophthalmoscope, etc.); these were frequently available in all rooms used for outpatient activities (N = 65; 59.1%). Most participating units (N = 79; 71.8%) had a dedicated physiotherapy and psychomotor rehabilitation room, and all had a specific room for speech and language therapy. Clinical and neuropsychological tests were available in nearly all the units (N = 107; 97.3%). An electronic clinical database was available in 94 units (85.5%), and the paper database met Italian privacy law requirements in 78 (70.9%) units. Online access to major scientific journals was available in 108 (98.2%) units.

### Outpatient CAMHS Staff and Functioning

As shown in table 1 the 110 participating units employed 769 full-time equivalent professionals i.e., 125 child psychiatrists, 147 clinical psychologists, 217 speech therapists, 13 psychomotor therapists, 125 physiotherapists and 118 educators. The CAMHS mainly employed permanent staff, and temporary contracts were quite infrequent (N = 67; 8.7%).

**Table 1:** Full-time-equivalent (FTE) CAMHS Professionals

	CAMHS full time equivalent professionals		N target population (0-17)/N professionals		N users/N professionals		Number professionals/100.000 target population (0-17)	Number professionals/100.000 total population
	**mean (sd)**	**Range**	**Mean (sd)**	**range**	**Mean (sd)**	**range**		

**Child psychiatrists**	11.19 (8.01)	4-29	5.807 (1152)	4.128-8.254	343 (91)	233-488	19.7	3.09

Psychologists (* = 1)	13.3 (8.25)	0-27	4.507 (1577)	2.727-8.128	260 (85)	161-433	23.2	3.64

**Speech therapists**	19.41 (10.40)	6-41	3.121 (583)	2.199-3.962	183 (41)	129-255	34.2	5.37

**Physiotherapists**	11.38 (7.32)	2-28	6.150 (2809)	3.075-11.843	352 (144)	193-700	19.7	3.09

Educators (* = 3)	10.75 (9.86)	0-29	5.974 (3056)	2.800-11.352	364 (205)	145-745	18.6	2.92

Psychomotor therapists (* = 5)	1.17 (1.42)	0-4	36.699 (19972)	10.464-68.645	2.028 (1099)	469-3.655	2.05	0.32

* Number of CAMHS without this health professional category

Staff supervision was available in 71 units (64.5%), and specific burnout prevention programmes were conducted routinely in only a few units (n = 9; 8.2%).

Most units were open Monday through Friday until 6 p.m. (N = 95; 86.4%), and 1/3 were also open on Saturday mornings (N = 32; 29.1%), with 39.35 (± 11.27) weekly mean opening hours.

All units granted direct free access, even without paediatric referral. The costs of clinical assessment (as with most clinical interventions), was fully covered by the National Health Service; a small fee was charged only by specific services, depending on the user's age and disorder.

On average, one-third of the units granted a first-visit appointment within a range of 15 days to 1 month (N = 37; 33.6%), whereas in another third, the delay was over 2 months (N = 28; 25.5%); a minority of units scheduled first visits within 7-15 days (N = 7; 6.4%).

A specific protocol for emergency 24-hr referral (priority of emergency consultation over ordinary scheduled visits) was present in 35 (31.8%) units. No CAMHS had a child psychiatrist on duty during night hours or holidays, and patients requiring treatment at these times were referred to ordinary E&A Departments.

In two third of the units (n = 74; 67.3%), first visits were conducted either by a neuropsychiatrist or psychologist, depending on staff availability, and in 18 units (16.4%) the neuropsychiatrist was the professional in charge of first visits.

Diagnoses were formulated according to the International Classification of Diseases, tenth edition [4] and were usually based on a detailed clinical interview of the patient and his/her parents.

Most interventions included counselling, individual rehabilitation training, and meetings with school teachers (table 2). Unconventional interventions, such as Pet Therapy and Music Therapy, were rarely practised (in 7 and 6 participating units, respectively).

**Table 2:** Range of CAMHS activities and procedures (*)

	N (%)
**Delivered treatments**	
• Meetings with Schools for disabled children	110 (100%)
• Counselling	100 (90.9%)
• Individual rehabilitation training-social skills training	81 (73.6%)
• Group rehabilitation training	64 (58.2%)
• Monthly meetings with welfare agencies	60 (54.5%)
• Psychoeducation	56 (50.9%)
• Consultation to paediatric firs aid	39(35.5%)
• Parent training	26 (23.6%)
• Art therapy and labs	23 (20.9%)
• Cognitive behavioural group therapy	19 (17.3%)
• Psychoanalytic group therapy	14 (12.7%)
• Pet therapy	7 (6.4%)
• Music Therapy	6 (5.5%)

**Availability of consultations with professionals from other units **(neuroradiologists, geneticists...)	91 (82.7%)

**Cooperation protocol with adult mental health services**	103 (93.6%)

**Frequency of staff meetings**	
• weekly	29 (26.4%)
• biweekly	36 (32.7%)
• monthly	26 (23.6%)
• less frequently	19 (17.3%)

**Protocol on communication of diagnosis to family members**	75 (68.2%)

**Cooperation protocol with paediatricians for the treatment of specific disorders**	
• Pervasive developmental disorder	30 (27.3%)
• Language disorders	13 (11.8%)
• Attention Deficit/Hyperactive Disorder	22 (20.0%)
• Eating disorders	21 (19.1%)
• Chronic and disabling disorders	15 (13.6%)

**Consent form for the use of psychotropic drugs**	69 (62.7%)

**Cooperation protocol with schools for special education needs**	86 (78.2%)

**Cooperation protocol with welfare agencies for abuse or family neglect**	67 (60.9)

(*) Information on the number (%) of units applying procedures

All CAMHS activities were documented in clinical records and frequently followed specific protocols; only a minority of the participating units shared treatment protocols with patients' paediatricians.

Most units had at least one board-certified psychotherapist; most of these had a psychodynamic training background (78 units; 70.9%), whereas systemic family psychotherapy was practised in 33 units (30%), and cognitive-behavioural therapy in 23 units (20.9%).

### Outpatient CAMHS activity data

**Figure 1:**
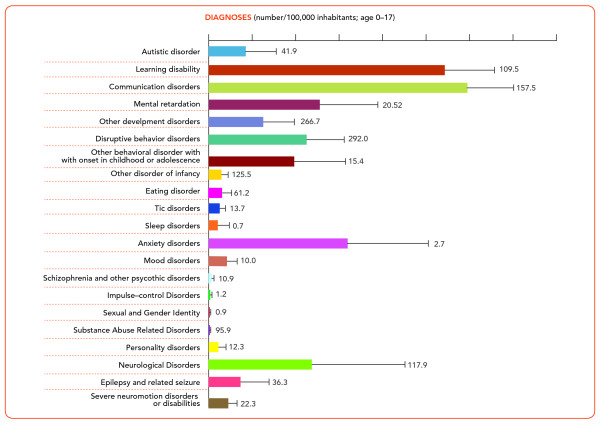
**Distribution of all diagnoses in the target population (0-17 years)**. Data refer to number of patients in treatment per 100,000 inhabitants aged 0-17 years, with standard deviations.

As show Figure 1, the 110 units evaluated and treated the entire range of mental and behavioural disorders with onset during childhood or adolescence. Most patients contacting CAMHS for the first time during the year 2008 received a diagnosis of communication or learning disorders. It is important to note that all diagnoses recorded on the regional registry were clinical diagnoses and were not obtained via standardised assessment methods.

**Figure 2:**
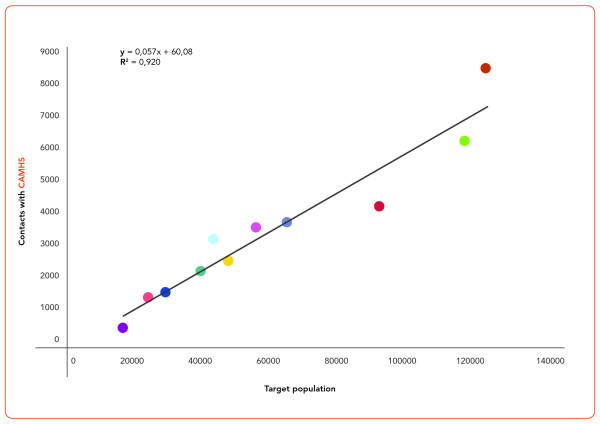
**Scatterplot of target population versus population in contact with CAMHS; strait line shows a linear regression curve**.

As show Figure 2, The average number of target population (0-17 years), for each CAMHS, was 5,947 ± 1,239 (range: 3,915-8,254), with almost perfect symmetry of distribution (Fisher skewness = 0.017); and moderate dispersion (CV = 0.21). Overall, approximately 6% of the 0-17 year target population was in contact with CAMHS in the Emilia-Romagna Region in 2008.

**Figure 3:**
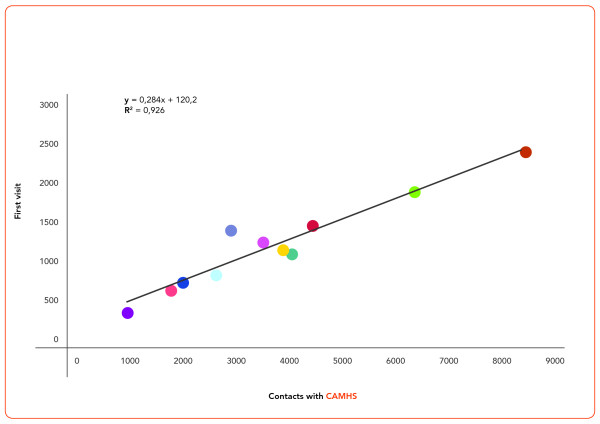
**Scatterplot of linear correlation of first visits number versus contact with CAMHS; strait line shows linear regression line**.

As show Figure 3, the average number of first visits during 2008 was 1,134 (± 604.3). First visit proportions were similar across the 11 CAMHS, accounting for 30% of their total annual child psychiatric consultations.

On average, in the 11 CAMHS 3,561 ± 2,042 child or adolescent patients had at least one contact in 2008, for an average of 6,313 ± 5,435 annual visits. Each patient received on average 2 or more visits by child neuropsychiatrists at that year.

### Inpatient psychiatric facilities for children and adolescents

The Emilia-Romagna Region had seven inpatient facilities (all public) for children and adolescents with neurological or behavioural problems; two were University Clinics, and one was a day-hospital.

With respect to environmental characteristics, the oldest facility was built in 1930, and all had been restructured over the previous ten years. Patients were generally hosted in double bedrooms; no single rooms were available.

Five inpatients units had dedicated rooms for clinical activities and meetings with families, and also had dedicated outdoor areas for patients (and their families).

In terms of organisational procedures, one facility admitted only patients with eating disorders; the others were generic units admitting any patients of paediatric age with mental disorders. All the facilities included a day-hospital.

Six inpatients units had inclusion/exclusion criteria for admission: three units did not admit patients with substance-related disorders, elevated suicide risk, or severe behavioural problems with aggressiveness, whereas all units treated severe MR or severe physical disabilities (e.g. blindness, paralysis...).

One inpatient unit's entrance was frequently kept locked, but all the other units had an open-door policy.

The amount of time available for family member visits was not standard: one unit permitted only two-hour family visit per day, but the other units allowed family members to stay all day.

Only two inpatient unit had a maximum hospital stay duration (30 days).

In terms of personnel, the inpatient units employed 29 child-psychiatrists, 17 (58.6%) of which were permanent staff. Treating staff included a further 68 professionals, mostly nurses, 30 of whom (44.1%) were permanently employed. The organisation of care during night shifts varied greatly by inpatient unit.

We also collected inpatient activity data for the year 2009: thirty-two (32) beds were available for the inpatient treatment of mental disorders, i.e., 5 "psychiatric beds" per 100,000 inhabitants aged 0-17 years. It should be noted, however, that these facilities can also admit patients from other regions; this proportion should therefore be interpreted with caution.

Overall, there were 624 admissions for the inpatient treatment of mental disorders in 2009, for a total of 7,822 hospital stay days. Among the 591 patients admitted in 2009, 411 (69.5%) were at their first ever admission. Moreover, a total of 1,038 patients were treated in day-hospitals in 2009 for a total of 1,046 admissions.

## Discussion

In Europe many studies have been conducted in the area of mental health services research; even in Italy, in the last 10 years, nationwide surveys have been conducted and they have provided valuable information on the characteristics, functioning and quality of services [5-13].

On the contrary, the situation in the area of child and adolescent mental health care is very different, and to our knowledge, this is the first study which provides detailed, updated and comprehensive health services research data on inpatient and outpatient services.

To our knowledge, the only study to date on this topic was conducted in England and Wales, to assess the distribution and few key characteristics of psychiatric inpatient units [14]. The only other studies available on mental health services for children and adolescents have focused mostly on costs [15], historical functioning and staff data [16], inpatient care effectiveness [17,18], service utilisation and provision [19,20], treatment intensity [21], and on determinants of unmet needs [22]. Although these studies have investigated relevant domains of mental health care for children and adolescents, none of them yielded quantitative data or provided detailed information on facility characteristics, staffing and patterns of functioning.

### Environmental characteristics and technical equipment of CAMHS

Overall, the quality of CAMHS logistic and architectural characteristics were found to be quite good, with an important exception, however. For example, most units were hosted in a single building with other public health services and only a few units had a separate, dedicated entrance. This can poses a crucial access problem-especially for units hosted within adult mental health services, which young patients and their families may want to avoid due to the stigma associated with facilities serving severe adult patients affected by chronic disorders. This facility characteristic may therefore represent a serious obstacle to the care-seeking process. Recent guidelines aimed at enforcing early intervention care protocols, emphasise the need for user-friendly settings, which better appeal to young patients requiring treatment and their families [23,24].

The majority of facilities were well equipped in terms of space and materials for both assessing and entertaining young patients. Electronic recording of basic clinical information was available in 85% of the units--a procedure that facilitates clinical evaluation and information sharing when needed. Most units complied with legal requirements on privacy issues.

### CAMHS staff and functioning

The number of child psychiatrists reported herein refers only to full-time equivalent physicians working in public mental health services. Child- and adolescent psychiatrists working exclusively in private practice were not included, although we note that their actual number might be not marginal. Our data showed that 3.1 child psychiatrists were available per 100,000 inhabitants, and specifically, 19.7 child psychiatrists per 100,000 inhabitants aged 0-17 years.

In the USA, the rate of child psychiatrists per 100,000 children-adolescents varies from 3.1 in Alaska to 21.3 in Massachusetts, but it is not known whether these numbers refer to full-time or part-time practice [25]. Moreover, child psychiatrists in Italy also treat neurological disorders, as their medical training covers both specialities. It is therefore not possible to disentangle the amount of time Italian child psychiatrists devote to the treatment of psychiatric and neurological disorders, and these data should be compared with data from other countries with caution.

In Europe, the rate of child psychiatrists per 100,000 inhabitants in the year 2007 ranged from a low of 0.3 in Bulgaria to a high of 3.5 in Sweden, but data on major countries, such as Belgium, Spain, Portugal, and England are currently lacking [26]. Moreover, the EUROSTAT report does not provide information on the methods employed to retrieve the data reported therein, and in Italy, reliable past information on this topic is unavailable.

In line with the percentage of the case-mix, speech therapists represented the highest proportion (16%) of staff, and their number was approximately double that of other professional categories (e.g. child psychiatrists, psychologists, physiotherapists, and educators).

Direct free CAMHS access was granted in all units, even without paediatrician referral. Yet, waiting times for first visits varied greatly across units, and only few made appointments within 15 days. Emergency referral was poor: the units had no staff on duty at night, and emergency referral 7/24 protocols were operative in only one third of the units. As shown by Worral et al., [27], this finding represents a main limitation for young patients with emergency needs, who end up receiving inappropriate interventions (e.g. by paediatricians or in adult mental health services).

### Diagnostic and treatment procedures

One major drawn-back in the functioning of Italian CAMHS is their lack of standardised assessment procedures, and diagnostic procedures are based on unstructured clinical evaluations. Similarly, outcome measures are not routinely applied, making demonstration of service quality and cost-effectiveness, and especially meaningful comparison, very difficult.

Moreover, although Italian CAMHS are also responsible for prevention, only a minority of the participating units shared specific protocols with paediatricians. Moreover, contrarily to current approaches in England [24] and Australia [28], early intervention services in Italy are still uncommon.

Lastly, Italy has no dedicated system of care available for children and adolescents who abuse drugs and/or alcohol. Users in young age are referred for assessment and treatment to standard addiction services attended by adult users. This state of affairs is surprising, considering that Italy does have dedicated Courts and prisons for individuals committing crimes under the age of 18 years. Yet, no targeted care and rehabilitation programmes are available for this vulnerable population.

### Activity data

Considering both mental and neurological disorders, communication disorder- and learning disabilities made up approximately half of the entire service user case-mix in 2008, whereas mood disorders were uncommon and accounted for only 1.5% of the sample. This study surveyed only public services, but in Italy, patients with minor anxiety or depressive disorders are more likely to be treated by private specialists (e.g., child psychiatrists, psychologists, and psychotherapists), which may in part explain the low prevalence of patients being treated for depression.

The main diagnosis prevalence showed a wide range of variability across the eleven CAMHS. Specialised services dedicated to specific disorders (e.g., learning disorders or autistic disorders) were available in some areas only; this situation may be linked to the higher prevalence of some disorders in some areas vs. areas lacking dedicated services. We also cannot exclude that across-unit assessment procedure differences, as well as different referral systems across CAMHS might have contributed to this prevalence data variability.

In addition to learning- and communication disorders, anxiety and disruptive behaviour disorders represented an important proportion of the CAMHS case-mix, i.e., 11.8% and 7.5%, respectively. Both conditions can be symptomatic of more severe and enduring disorders, and can even represent an ongoing-psychosis symptom profile [29]. Yet, in Italy dedicated early assessment and intervention protocols for psychosis--for both the schizophrenia and bipolar spectra--are rarely available, despite increasing attention being focused on this issue worldwide [30]. CAMHS case-mix examination, however, is generally rare; with the exception of one Norwegian study, which recently analyzed reasons for referral to CAMHS and found that sadness or depression accounted for 19% of patients in contact [21].

We observed a homogeneous proportion of patients in contact with CAMHS (approximately 6% of the targeted population aged 0-17 yrs.), a finding in line with previous epidemiological Italian studies [3]. Prevalence studies in other countries have shown that up to 30% of children and adolescents suffer from a mental disorder, and half of those presenting current psychopathology suffer from severe impairment [31]; current figures about treatment rates, however, show that even in the area of child mental health there is a substantial treatment gap between children and adolescents who suffer from disorders and those who actually receive any kind of care [32]. In Italy, the prevalence of mental disorders among children and adolescents appears to be lower than in other countries (8%), and this finding is congruent with the lower prevalence of mental disorders also observed in the adult population [33]. Yet, the rate of 8% found in the only Italian study conducted with methodological rigor [3] did not include language and learning disorders, which conversely represent the largest share of disorders treated at the CAMHS surveyed in the present study. We therefore assume that, even in Italy, there is a a substantial treatment gap [32].

Both the proportion of young people in contact with services (approx. 6%) and the proportion of first visits (30%) with respect to the annual visit total were homogeneous across the eleven CAMHS. This finding shows a rather uniform across-CAMHS distribution of resources, in terms of availability of professionals and location of facilities. At the same time, however, these data also suggest that all CAMHS are already treating the maximum number of patients in function of their available resources, and thus, that no more than 30% of the total consultations can be reserved to first visits.

Concerning inpatient facilities, a nationwide study conducted in England [14] found a range of available beds throughout the nation (3.4-12.9 beds per 100,000 population aged 18 yrs. and younger) which was close to the rate observed in the present study (5 per 100,000). In Italy, however, patients younger than 18 years and suffering from severe disorders may also be admitted to GHPUs if necessary, but this approach represents a main shortcoming in the mental health policy, due to the inadequacy of many adult inpatient services for a paediatric population.

It would be interesting to provide an esteem of the CAMHS costs because these information might be useful for planning purposes and for comparison with other countries. In Italy there have been a few detailed studies on costs of mental health care [34-36]. However there have been no studies about the specific costs of CAMHS, as done in other countries [15,17,37]. In the Emilia-Romagna Region, in a 2010 report [38], it is stated that the cost of all mental health care delivered through DMHs was 4.7% of all health expenditures for that year; this figure includes hospital, outpatient and residential care. Since DMHs deliver all mental health care, including both adult and child-adolescent populations, it is impossible to disentangle this figure from the specific costs of CAMHS. This is an area which deserves additional efforts in Italy.

### Limitations and strength of the study

The present is a cross-sectional study based on interviews with service staff: diagnosis data therefore could not be checked with standardised instruments. Although the study was limited to one large Region of the country, it is likely that the characteristics of CAMHS of this Region are very similar to those of other regions of the North and Centre of Italy, which share very similar socioeconomic and cultural characteristics with the Emilia-Romagna Region, as well as a similar architecture of health services.

## Conclusions

With these limitations in mind, the present study shows that CAMHS are distributed uniformly throughout a large Italian region. The overall number of child psychiatrists per 100,000 inhabitants is one of the highest in Europe and is comparable to the best-equipped areas in USA. Although their logistic and technical characteristics appear satisfactory, future research efforts should address to the day-to-day functioning of these services.

One main priority emerging from this survey is the need for standardised assessment procedures for both diagnostic and outcome-evaluation purposes to be introduced in these services. Another important issue is that of poor focus on preventive and early intervention observed herein: most major mental disorders have their onset in adolescence, and early assessment and intervention targeting young people has become a worldwide priority.

## Competing interests

The authors declare that they have no competing interests.

## Authors' contributions

LP, GdG, AP have conceived the study, have selected and developed the assessment instruments, have organized and supervised data collection, were actively involved in data analyses and have drafted the paper. G.C., SC, MF, LF, EF, FM, RM, VN, SP, RP, AP, CR, AS, ST, DT, PV, AT have made substantial contributions to the conception of the study and to the development and validation of instruments, have organized and supervised data collection in each catchment area, and gave comments on the various drafts. DS and MBLR have carried out the statistical analyses. All authors have read and approved the final manuscript.

## Pre-publication history

The pre-publication history for this paper can be accessed here:

http://www.biomedcentral.com/1471-244X/12/7/prepub
